# Complete Genome Sequence of the Livestock-Associated Methicillin-Resistant Strain *Staphylococcus aureus* subsp. *aureus* 08S00974 (Sequence Type 398)

**DOI:** 10.1128/genomeA.00294-17

**Published:** 2017-05-11

**Authors:** Olga Makarova, Paul Johnston, Birgit Walther, Jens Rolff, Uwe Roesler

**Affiliations:** a Institute of Animal Hygiene and Environmental Health, Centre for Infection Medicine, Freie Universität Berlin, Berlin, Germany; b Evolutionary Biology, Institute of Biology, Freie Universität Berlin, Berlin, Germany; c Berlin Center for Genomics in Biodiversity Research, Berlin, Germany; d Leibniz-Institute of Freshwater Ecology and Inland Fisheries (IGB), Berlin, Germany; e Institute of Microbiology and Epizootics, Centre for Infection Medicine, Freie Universität Berlin, Berlin, Germany

## Abstract

We report here the complete genome sequence of the livestock-associated methicillin-resistant *Staphylococcus aureus* strain 08S00974 from sequence type 398 (ST398 LA-MRSA) isolated from a fatting pig at a farm in Germany.

## GENOME ANNOUNCEMENT

*Staphylococcus aureus* subsp. *aureus* strain 08S00974 is a methicillin-resistant member of sequence type (ST) 398. This livestock-associated clonal lineage, which first became known for its widespread colonization of industrially raised pigs (1), exhibits an extended host spectrum genotype (2). A broad host range in combination with a continuous acquisition of virulence and antibiotic-resistance genes from hospital-associated clones of *Staphylococcus aureus* make the members of this sequence type a serious threat to public health (3). To aid the studies of host adaptation and antimicrobial resistance, we sequenced the complete genome of *S. aureus* subsp. *aureus* strain 08S00974. This strain was originally isolated from a fatting pig raised at a farm in Germany and has been used for host colonization kinetics and susceptibility studies (4).

The reads were obtained using a PacBio RS II sequencer using P6/C4 chemistry (10-kb insert library; average read length, 93.93 bp; 289,303 reads) and an Illumina MiSeq (TruSeq DNA PCR-free library; 300-bp paired-end reads; 518,176 read pairs). A single contig was produced during the *de novo* assembly using Canu (5) and circularized using Circlator (6). Alignments produced by mapping Illumina reads to the assembly using the Burrows–Wheeler alignment method (7) were used to correct errors with Pilon (8). The resulting complete genome was annotated using Prokka (9).

The ST398 08S00974 chromosomal genome is 2,849,409 bp (G+C content, 32.9%) and possesses 2,551 open reading frames, 19 rRNAs, and 62 tRNAs. Prophage sequence prediction server PHAST (10) identified one intact 73.6-kb prophage sequence in the region between 1,830,776 bp and 1,904,406 bp, which bears similarity to the staphylococcal phage StauST398-2 (NC_021323). The strain belongs to ST398, *spa*-type t011, and SCC*mec* type V, according to sequence typing analysis using Ridom SeqSphere+ (Ridom GmbH, Germany) (11). The strain harbors a complete beta-lactamase operon (*bla*Z, *bla*I, *bla*R), genes associated with resistance phenotypes toward tetracycline (*tet*K, *tet*M), and licomycin /streptogramin (*vga*A). Genes associated with the immune evasion cluster and exfoliative toxins described so far were not detected.

### Accession number(s).

The complete genome sequence of *S. aureus* subsp. *aureus* strain 08S00974 has been deposited in GenBank under the accession number CP020019.
